# Comparison of Respondent Driven Sampling Estimators to Determine HIV Prevalence and Population Characteristics among Men Who Have Sex with Men in Moscow, Russia

**DOI:** 10.1371/journal.pone.0155519

**Published:** 2016-06-01

**Authors:** Andrea L. Wirtz, Shruti H. Mehta, Carl Latkin, Carla E. Zelaya, Noya Galai, Alena Peryshkina, Vladimir Mogilnyi, Petr Dzhigun, Irina Kostetskaya, Chris Beyrer

**Affiliations:** 1 Center for Public Health and Human Rights, Department of Epidemiology, Johns Hopkins Bloomberg School of Public Health, Baltimore, United States of America; 2 Department of Epidemiology, Johns Hopkins Bloomberg School of Public Health, Baltimore, United States of America; 3 Department of Health, Behavior and Society, Johns Hopkins Bloomberg School of Public Health, Baltimore, United States of America; 4 Department of Statistics, The University of Haifa, Mt Carmel, Israel; 5 AIDS Infoshare, Moscow, Russian Federation; David Geffen School of Medicine at UCLA, UNITED STATES

## Abstract

Analytically distinct estimators have been proposed for the calculation of population-based estimates derived from respondent-driven sampling (RDS), yet there have been few comparisons of the inferences from these estimators using empirical data. We compared estimates produced by unweighted analysis used to calculate sample proportions and by three available estimators that are used to calculate population proportions, RDS-I, RDS-II (Volz-Heckathorn), and Gile’s RDS-SS. Data were derived from a cross-sectional, RDS study of men who have sex with men (MSM) conducted from October 2010 to April 2013 in Moscow, Russia (N = 1,376, recruitment depth: 31 waves). Analyses investigated the influence of key parameters: recruitment depth, homophily, and network size on sample and population estimates. Variability in results produced by the estimators and recruitment depth were statistically compared using the coefficient of variation (CV). Sample proportions had the least variability across different recruitment depths, compared to the RDS estimators. Population estimates tended to differ at lower recruitment depth but were approximately equal after reaching sampling equilibrium, highlighting the importance of sampling to greater recruitment depth. All estimators incorporate inverse probability weighting using self-reported network size, explaining the similarities in across population estimates and the difference of these estimates relative to sample proportions. Current biases and limitations associated with RDS estimators are discussed.

## Introduction

Respondent-driven sampling (RDS) has emerged as a common method in HIV research to recruit hidden populations including men who have sex with men (MSM), sex workers, and people who inject drugs.[1, 2] These groups may be criminalized, stigmatized, difficult to reach, and generally lack sampling frames needed for probability-based sampling methods.[3] RDS attempts to overcome these limitations through two innovations: providing a means to reach hidden populations through peer networks and generating population estimates using specialized estimation methods.

RDS recruitment begins with ‘seeds’—well-networked individuals from the target population who are purposively selected and represent a range of characteristics. Seeds participate in study activities and are asked to recruit a limited number of peers to participate. Recruited peers who participate, in turn, become recruiters and are requested to invite a new selection of peers to participate. This process continues through successive waves of recruitment until the target sample size is reached.[4] The reliance on peer trust, allowance of limited peer recruitment by each participant, and use of double incentive system, in which participants are incentivized for both participation in the study and for recruitment of peers, aid in driving recruitment further into the target population than would typically be achieved through convenience or snowball sampling techniques.[2]

The second benefit of RDS is the ability to generate population estimates based on the underlying network from which the sample was derived. Estimation methods build on Markov chain theory and assume that after several waves of recruitment the total sample is no longer influenced by the initial sample (seeds) and begins to represent the underlying population.[5] Inherent in this are several assumptions that influence either recruitment or inference. With respect to statistical inference, assumptions include: 1) Sampling is with replacement, in which selected peers may be recruited multiple times; 2) Network size: that participants can accurately report personal network size; 3) Random recruitment: peer recruitment is a random selection from recruiter’s network.[6] Despite widespread use of RDS, these conditions are often not be met in practice. Most research relies on sampling without replacement, preventing individuals from participating more than once. Additionally, it is not known how accurately individuals can report their network size within the target population. Finally, secondary incentives may reduce the randomness of peer recruitment as participants preferentially recruit peers based on their relationship and/or assumptions of who will participate.[7]

Several estimators have been proposed to produce theoretically unbiased population-based estimates for RDS data. One of the earlier estimators, RDS-I, developed by Salganik and Heckathorn (also called the Salganik-Heckathorn estimator) incorporated the use of referral patterns, network size, and the number of cross-relation ties between subgroups of interest.[8, 9] A standalone software, Respondent Driven Sampling Analysis Tool (RDSAT), was developed that included RDS-I, leading to widespread use of this estimator.[10] RDS-II, the Volz-Heckathorn (V-H) estimator was subsequently proposed as an improvement upon and address biases associated with RDS-I.[11] The RDS-II estimator, like RDS-I, relies on sampling probabilities and incorporates individual degree, or network size, to create sampling weights, but does not rely on cross-group relations.[11] Schonlau and Liebau later developed a package for the implementation of RDS-I and RDS-II within Stata statistical software.[12] To address concerns related to the use of sampling without replacement in RDS studies, Gile and colleagues developed the RDS successive sampling estimator (RDS-SS) and a corresponding implementation package, RDS Analyst (RDSA) for use in R, which also includes RDS-I and RDS-II estimators.[13, 14]

Despite on-going improvements in these estimators, debates continue on how accurately these estimators capture true population values and to-date there is no consensus on which estimator is optimal.[15] Among hidden populations the true population values are difficult, if not impossible, to measure, so validation of population estimates has been explored in more general population samples. McCreesh and colleagues carried out an RDS survey in a community in Uganda to compare sample, RDS-I, and RDS-II estimates to known populations. The authors found that only 31–37% of the population estimates were closer to the true population proportions, compared to the sample proportions. In some cases, sample estimates more accurately captured the true population values.[16] Other concerns relate to the potential biases associated with the practice of sampling without replacement and Gile and Handcock add that RDS-II is sensitive to preferential referral among participants.[17] Other sensitivities of RDS demonstrated by simulation studies include biases induced by errors in self-reported network size and biases induced by the initiation sample (seeds).[13, 18]

In practice, reporting of RDS data has been inconsistent. Often, sample proportions are not reported, nor are recruitment depth or total sample size, limiting critical review or reanalysis of data.[19] There is no accepted convention on which estimator should be used, nor whether sample estimates (unweighted) or population estimates (RDS-weighted) or both should be presented. The reason for estimator selection is often absent or vague in descriptions of methods; [19] in many cases, selection may be based more on preference for a statistical software than consideration for estimator itself. General understanding of the use and sensitivities of RDS comes from statistical simulations with less frequent examination of the variability of RDS estimators in field research.

The analysis presented here is derived from a study of HIV among MSM in Moscow, Russia where RDS was used as a means to recruit hidden populations of MSM into an epidemiologic study and engage MSM in safe HIV testing opportunities. This analysis aimed to demonstrate the performance of the RDS estimators in population inference of HIV prevalence and behavioral characteristics as they relate to natural variations of key parameters within the empirical dataset.

## Methods

Data were collected as part of a cross-sectional study to characterize the HIV epidemic among gay, bisexual, and other MSM from October 2010 and April 2013. Details of the sample, study site, recruitment, and measurement methods have been described elsewhere.[20, 21] To protect the confidentiality of participants, all participants provided oral consent to participate in the study activities and all data were anonymously collected. The study was approved by the Ethics Committee of the State Medical University, IP Pavlov, St. Petersburg, Russia and The Johns Hopkins Bloomberg School of Public Health Institutional Review Board, Baltimore, Maryland.

### RDS-recruitment

Recruitment began with 14 purposively selected seeds. These individuals were recruited in a staggered fashion over the course of the study and were given four study coupons with which to recruit peer MSM. Eligible and participating peers were in turn provided with three study coupons for peer recruitment and this process was repeated until the target sample size of 1,370 was reached.

### Measures

Participation in the study included a sociobehavioral survey and biological assessment of HIV and syphilis. All participants were asked personal network size questions used for RDS weighting (“How many people do you know personally who are men who have sex with men, live in the Moscow region, and who are 18 years old or older?” and “Of these men, how many have you seen in person at least once in the last 6 months?”).[3, 4]

Survey measures included demographic characteristics; sexual identity and relationships; substance use; and depression symptoms. Key variables for this analysis were selected to capture demographic, sexual identity and behavior, and infectious disease domains. We present the results pertaining to four variables: self-reported country of birth, sexual identity, consistent condom use during anal intercourse, and HIV infection. These variables were selected to represent high or low homophily, differences in average network size among variable subgroups, and included both self-reported characteristics and clinically determined outcomes. Country of birth was defined as being born in or outside of Russia. Sexual identity included three categories: homosexual, bisexual, or heterosexual/other. Condom use consistency during anal sex (last 6 months) was a binary self-reported measure: inconsistent (use of condoms only half the time, rarely, or never) and consistent condom (always or almost always). HIV infection status was determined via OraQuick Rapid HIV 1/2 test (OraSure Technologies, Bethlehem, PA, USA) and either confirmatory testing at the local reference laboratory (Lages Laboratory, Moscow) or self-reported HIV infection without confirmatory testing. Participants were offered HIV testing, but were allowed to decline testing and still participate in the survey, resulting in a greater response rate for the other variables included in the analysis.

### RDS-estimators and analysis

Sample proportions without sampling weights were calculated for each variable. Population (RDS- proportions weighted) were separately calculated using the RDS-I, RDS-II, and RDS-SS estimators. Seeds were excluded from all analyses. RDS-I and RDS-II (Volz-Heckathorn) population proportions were calculated using weights derived from individual self-reported network size. Unlike RDS-I, RDS-II does not rely on the use of recruitment chains for estimation, though all statistical packages still require the underlying recruitment structure for estimation.[10, 12, 14] RDS-SS also requires the input of the population size.[13] For unknown MSM populations, an assumption of 1–3% of the adult male, general population is used to estimate the MSM population size.[13] An estimate in the range of 34,678–104,035 MSM in Moscow City was calculated assuming MSM represented 1–3% of the 3,467,836 adult men aged 18–54 residing in Moscow city in 2012.[22] The model assisted estimator (RDS-MA) has recently been proposed as a means to address seed bias that may be present in existing estimators; however, we did not examine its use given limited availability at the time of the analysis.[23]

Analysis of sample proportions, RDS-I, and RDS-II estimates were implemented in Stata 13 (College Station, TX) using the RDS statistical implementation package developed by Schonlau and Liebau.[24] Non-parametric bootstrapping of RDS-I and RDS-II estimates with 1,000 iterations was implemented to produce 95% confidence intervals (95%CI). Analysis of RDS-SS estimates was conducted using RDS-A within R software with 1,000 bootstrap iterations to produce 95% CIs.[14] To assess the sensitivity of the estimator to variation in population size, RDS-SS estimation was run twice using both population sizes that were based on 1% and 3% of the male population, rather than using the mid-point of the estimate, which is the RDS-SS default. RDS-I and RDS-II estimates produced by Stata with RDSA were also cross-checked to identify differences across software.

To assess the role of RDS parameters on sample and population estimates, homophily, recruitment depth, and average network size were estimated for each subgroup of the variables of interest. These parameters were calculated using the Stata implementation package.[12] Calculated homophily values can fall within the range of– 1 to 1, with estimates close to 1 indicating a strong likelihood to recruit from the same subgroup. Low homophily values are those that are close to 0, representing fairly neutral recruitment by one subgroup across other subgroups.[12] Variables with higher levels of homophily were selected for analysis of the role of homophily; these included country of birth and sexual identity. High levels of homophily range between 0.7–1.0; however, none of the variables in this data set were above 0.45, limiting the assessment of the influence of homophily. Recruitment depth is the number of waves of participants reached through RDS. RDS studies report typically reaching an average of about 6–10 waves of recruitment.[19] Convergence was also calculated for each variable and denotes the required recruitment depth necessary for a transition matrix to reach sampling equilibrium to the Markov process.[11] Individual network size is calculated by the number of other MSM the participants report knowing; these values are then averaged for each subgroup of each variable the individuals endorse.[12]

RDS estimators were compared using both graphical and statistical comparison methods. To analyze the role of recruitment depth (numbers of waves recruited), sample and population prevalence estimates were calculated for each variable of interest and recalculated when data were restricted to depths ≤5, ≤10, ≤20, and ≤31 waves, the last being the maximum recruitment of the sample. Estimates were plotted graphically with 95%CIs to compare the sample and population estimates of key subgroups of interest for each variable across specified recruitment depths. Graphs are displayed with Y-axis spanning a difference of 25% (i.e. they do not begin at 0%) for the purposes of displaying relatively small differences in estimates. The coefficient of variation was used to statistically compare the variability of estimates produced by the different RDS estimators.[25] CV was used to assess the variability in estimates produced by the RDS estimators in two forms: 1) the distribution of estimates produced with each estimator for each variable at full recruitment, and 2) the distribution of estimates produced at each specified recruitment depth across the RDS estimators.

## Results

Six of 14 seeds successfully propagated five or more waves of recruitment. One seed that was initiated early in the study recruited 74% of the sample. A total of 3,997 coupons were distributed, yielding 31 waves of recruitment with 1,429 recruits and an overall coupon return rate of 0.36. Of these recruits, 1,362 were eligible for the study (1,376 including seeds). Table 1 presents RDS recruitment process indicators and seed descriptions.

**Table 1:** RDS process indicators and seed characteristics in the recruitment of MSM in Moscow, Russia (2010–13).

	Seed ID					
	1	2	3	4	5	6
Number of waves per seed:	31	9	5	5	12	9
Number of persons recruited per seed:	1,048	41	25	25	137	120
Coupon return rate per seed:	0.353	0.357	0.410	0.595	0.365	0.368
Number of eligible participants per seed:	1,004	37	20	15	137	120
Characteristics:						
Age (years):	44	44	35	28	32	36
Country of birth:	Russia	Russia	Russia	Russia	Russia	Outside Russia
Sexual identity:	Homosexual	Bisexual	Bisexual	NR	Bisexual	Homosexual
Condom use:	Consistent	Consistent	Inconsistent	Consistent	Inconsistent	Consistent
HIV status:	Uninfected	NR	Uninfected	Infected	Infected	Infected

Note: NR: Not reported

Table 2 presents basic demographics of the study sample.

**Table 2:** Demographic and HIV-related characteristics among RDS-recruited MSM in Moscow, Russia (N = 1,376).

	n	Col %
Age categories		
<25	399	29.0
25–29	321	23.3
30–35	326	23.7
>35	330	24.0
Place of Birth		
Born in Russia	1164	84.6
Born outside Russia	212	15.4
Ever married to a woman (n = 1,368)		
Never	1128	82.5
Past/current marriage	240	17.5
Employment categories (n = 1,367)		
Full-time	744	54.4
Part-time	457	33.4
Student	61	4.5
Other	28	2.0
Unemployed	77	5.6
Income categories (n = 1,365)		
High	23	1.7
Middle	605	44.3
Low	692	50.7
Poverty	45	3.3
Location where participants normally go for healthcare (n = 1,253)		
Private only	354	28.3
Public and Private/Other	159	12.7
Public only	699	55.8
Other only	41	3.3
Sexual Identity (n = 1,347)		
Homosexual	741	55.0
Bisexual	578	42.9
Other	28	2.1
No. of Male Partners last 12 months		
1	341	24.8
2 to 4	373	27.1
> = 5	662	48.1
HIV status (by confirmatory test or rapid test with positive self-report; n = 1,173)		
Negative	995	84.8
Positive	178	15.2

Note: col. % represents column percent

### Recruitment Depth

**Fig 1:**
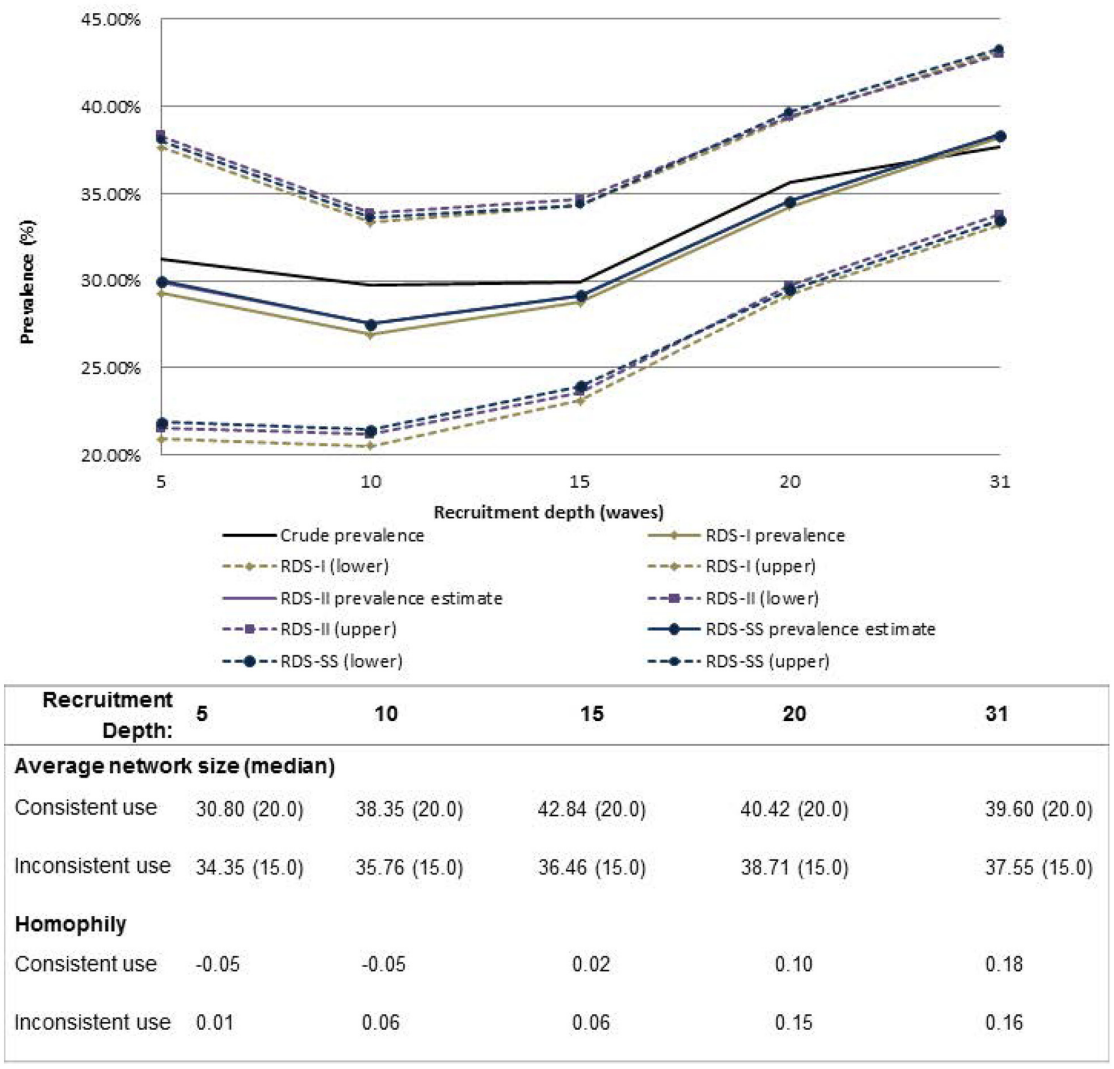
Sample and population proportion estimates of inconsistent condom use, by recruitment depth and estimation method. Table includes average network size and homophily at each recruitment depth; RDS-II and RDS-SS estimates overlap; dashed lines represent 95%CIsConsistently, the variability of population estimates produced at higher waves of recruitment tended to be lower compared to the variability of estimates produced at lower waves of recruitment (Table 3). For example, CV estimates for inconsistent condom use range from a high of CV_wave 5_ = 0.013 at recruitment depth ≤5 waves and drop to CV_wave 31_ = 0.003 by wave 31.

**Fig 2:**
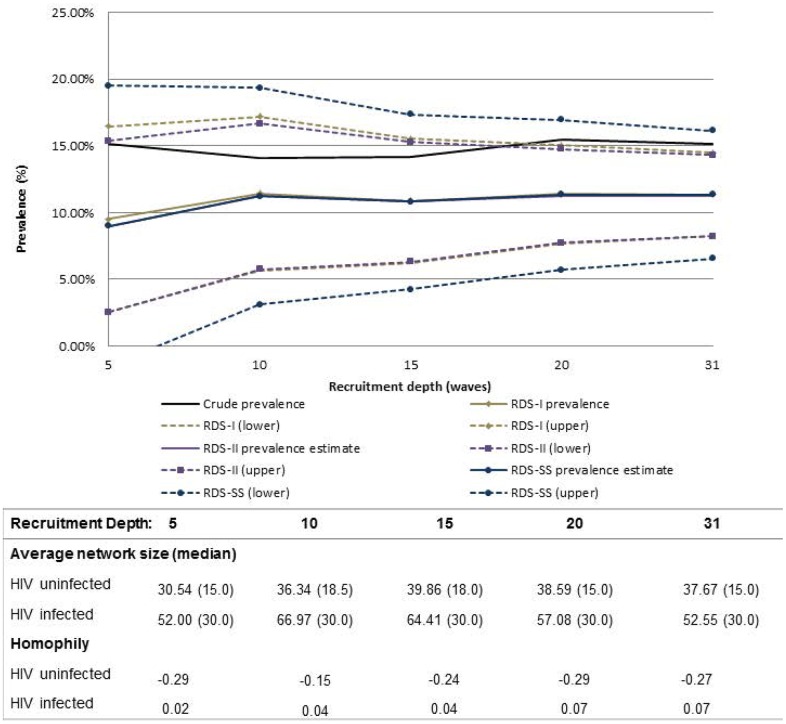
Sample and population proportion estimates of HIV infection, by recruitment depth and estimation method. Table includes average network size and homophily at each recruitment depth; with exception of the upper confidence interval, RDS-II and RDS-SS estimates overlap; dashed lines represent 95%CIs.

**Fig 3:**
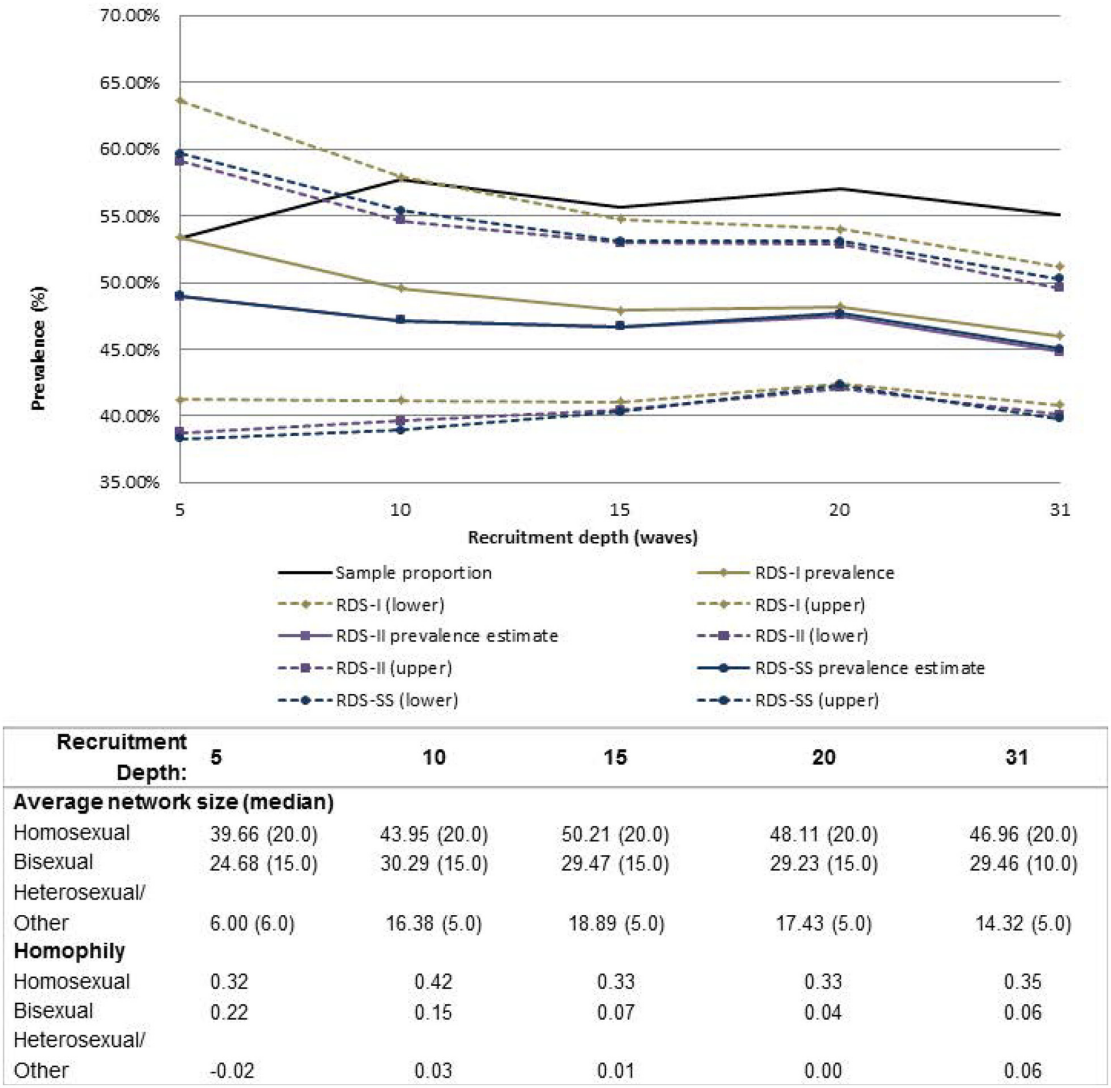
Sample and population proportion estimates of MSM with homosexual identity, by recruitment depth and estimation method. Table includes average network size and homophily at each recruitment depth; with exception of 95%CIs, RDS-II and RDS-SS estimates overlap; dashed lines represent 95%CIs.

**Fig 4:**
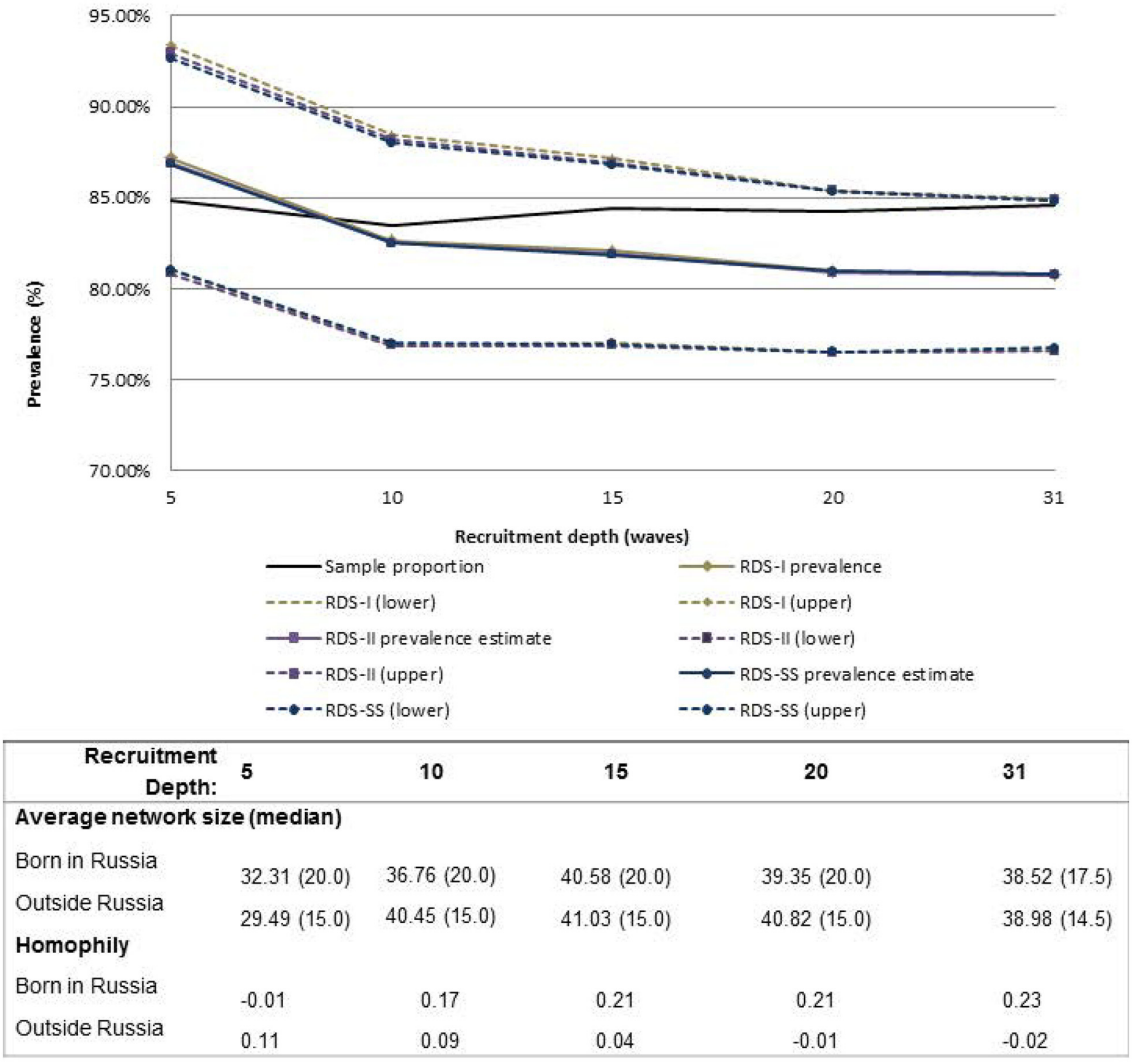
Sample and population proportion estimates of MSM born in Russia, by recruitment depth and estimation method. Table includes average network size and homophily at each recruitment depth; with exception of upper 95CIs, RDS-I, RDS-II, and RDS-S estimates overlap; dashed lines represent 95%CIs.

All convergence estimates reported to reach sampling equilibrium by a depth of 3 to 4 waves for each variable. For most variables, except inconsistent condom use, sample and population proportion estimates appeared to stabilize after 5–10 waves of recruitment (Figs 1–4). Sample proportions were higher than calculated population proportions for all subgroups depicted. This difference was found for estimates produced across almost all analyzed recruitment depths, with the exception of inconsistent condom use which crossed the RDS estimators above a recruitment depth of wave 30. At full recruitment (31 waves), the sample and population proportions for inconsistent condom use were approximately equal (Fig 1).

Sample proportions of all variables had the lowest variability in estimates produced across all variables. The variability in HIV prevalence estimates illustrates this case in which the CV_SP_ = 0.043, compared to higher variability among the other RDS estimators (CV_RDS-I_ = 0.074; CV_RDS-II_ = 0.093; CV_RDS-SS_ = 0.092; Table 3). Among the RDS estimators, however, variability was similar. All 95% confidence intervals became more precise with increasing sample size, which may be reflective of increasing sample size rather than a reflection of the estimator. However, at higher recruitment depths, the confidence intervals no longer captured the sample proportion for HIV infection and sexual identity (Figs 2 and 3).

**Table 3:** Variability of sample proportion and estimators for select variables, as measured by coefficient of variation across estimators and recruitment depth.

	Inconsistent condom use	HIV Infection	Born in Russia	Homosexual Identity
**Estimation method** ^1^	CV	CV	CV	CV
Sample Proportion	0.110	0.043	0.006	0.031
RDS-I	0.147	0.074	0.032	0.056
RDS-II	0.140	0.093	0.030	0.032
RDS-SS	0.140	0.092	0.030	0.030
**Recruitment depth** (numbers of waves reached) ^2^				
≤ 5	0.013	0.033	0.016	0.039
≤ 10	0.013	0.011	0.005	0.028
≤ 15	0.008	0.004	0.006	0.015
≤ 20	0.005	0.005	0.002	0.008
≤ 31	0.003	0.004	0.002	0.012

Note:

^1^ CV measured for sample proportion and each estimator within each variable at full recruitment (wave≤31);

^2^ CV of recruitment depth measured across RDS-I, RDS-II, and RDS-SS within each variable at specified recruitment depths

### Average Network Size

There was little difference in average network size for the two condom use subcategories across waves: at recruitment depths ≤5 waves, the average network size was 30.80 and 34.35 for consistent and inconsistent condom use, respectively, and increased to 39.60 and 37.55 by a recruitment depth of ≤31 waves (Fig 1). With generally low and similar network size across both subgroups, both sample and population proportions track closely together after about 15 waves (Fig 1). Contrary to the prior example, the average network size among HIV infected MSM was substantially higher than among HIV uninfected MSM, with average network size among HIV uninfected men estimated at 30.54 and 52.00 among HIV infected men at wave≤5. At recruitment depth ≤31 waves, the average network sizes for these two groups were 37.67 and 52.55, respectively (Fig 2). There are visible differences between the sample and population proportions for HIV infection in which estimates for HIV infected participants with higher network sizes are down-weighted (Fig 2). This is a similar case for homosexual identity (Fig 3).

### Homophily

Among variables with low homophily, inconsistent condom use and HIV infection, RDS estimators produced fairly similar results, though with greater variability at lower recruitment depth. This was most apparent for inconsistent condom use estimation when recruitment depth was equal to or less than 5 or 10 waves (CV_*wave5*_ = 0.013; CV_*wave10*_ = 0.013) compared to when recruitment depth was equal to or less than 20 and 31 waves (CV_*wave20*_ = *0*.*005*; CV_*wave31*_ = 0.003; Table 1). For HIV infection, population proportions produced by RDS estimators were approximately equivalent after 10 waves of recruitment and variance was substantially lower in later waves (CV_*wave5*_ = *0*.*033*; CV_*wave20*_ = 0.005; CV_*wave31*_ = 0.004; Table 3).

Similar results among the RDS estimators were observed for variables with higher homophily: homosexual identity and country of birth (homophily = 0.30–0.42; Figs 3 and 4). Estimated proportions of self-reported birth in the Russian Federation were approximately equal for all estimators, though with an intersection prior to wave 10 in which the RDS estimators began producing lower proportions relative to the sample proportions. In this example, homophily is low during early waves (-0.01) and increases to 0.21 in wave 15, but with likely little influence on the estimators due to the number of waves reached (Fig 4). For the variable of sexual identity, the RDS-I estimator, because of its reliance on cross-group relationships, overestimated the other RDS estimators until recruitment depth reached wave 20, in which all estimators began to produce similar results (Fig 3).

## Discussion

Beyond successful recruitment of hidden populations, use of RDS provides an additional innovation in providing inference to the target population through the use of weighting techniques. Past simulations have demonstrated reductions in bias with each new estimator that has been developed, suggesting that each estimator may produce different results.[5, 13] This analysis of data from a sample of MSM in Moscow, Russia, however, demonstrates little difference in results produced by the available estimators.[5, 13, 17] Differences in the RDS estimators tend to appear in early waves until estimates have stabilized, typically after 5 to 10 waves of recruitment, when seed bias is likely reduced. Greater variation during earlier phases of recruitment are most evident with the RDS-I estimator, which has been de-emphasized more recently as a method for weighting.[11, 17] Studies with low recruitment depth, either due to a small sample size or relatively large number of seeds, may take this into consideration when considering the type of estimator used and may consider continuing recruitment to extend chains to an appropriate depth.

Variability of the estimators at low recruitment depth may be explained by seed bias, in which non-random selection of seeds biases the population estimates.[17] It is worth noting, however, that concerns about seed bias and depth were derived from simulations of sampling depths with 4 or 6 waves.[17] A prime example in this study was the case for the country of birth variable, in which the higher proportion of seeds reporting birth in the Russian Federation (versus outside) appeared to bias the RDS estimates at low recruitment depth, prior to convergence to sampling equilibrium. With increased recruitment depth, these differences between the estimators tended to disappear, which is consistent with reports by Volz and Heckathorn in the original comparison of RDS-I and RDS-II.[11] While RDS-II is apparently more stable for analysis of studies with short recruitment chains, relative to RDS-I, it is unclear how RDS-II compares to RDS-SS for short recruitment chains. Early research on the use of RDS-MA, which is currently not in widespread use, has demonstrated that this estimator does account for the bias associated with seed selection and may be appropriate for such analysis in the future.[23] Despite demonstrated implications of seed bias, it is not recommended, however, to remove data from early waves to reduce seed bias, as simulations have demonstrated that the RDS estimators are robust against inclusion of out-of-equilibrium data (data produced by waves before sampling equilibrium has been reached) and removal of out-of-equilibrium data from analysis may potentially introduce greater bias.[17, 26]

RDS-I and RDS-II estimation methods are grounded in Markov chain theory and rely on the assumption of sampling with replacement. Because sampling with replacement is not generally practiced in survey research, these estimators then rely on a small sampling fraction so that the sampling with replacement solutions inherent in the estimators may be applied.[11] Therefore, it has been demonstrated that recruitment that results in a large sampling fraction can also lend to bias associated with the RDS-II estimator.[17] RDS-SS adjusts for this by considering the population size in the estimation process and may provide an improved alternative estimator in cases where the sampling fraction is large.[13] The disadvantage, however, is that RDS-SS requires knowledge of the population size, which is often not known for hidden populations or may be highly variable, depending on the estimation method(s) used.[13, 27] In this study, which was conducted in Moscow City, the sampling fraction is small and we observed little difference between RDS-II and RDS-SS. Surveillance and other research conducted in rural areas and low population density should be aware of potential limitations of recruiting a large sampling fraction in areas where the target population may be small.[17]. Consideration to appropriate recruitment depth and sampling fraction is important during the recruitment process as well as analysis.

Sample proportions, which are considered to be approximate to a convenience sample, tend to have the least variability across various recruitment depths. These proportions may be influenced by the recruitment process, in which well-networked participants may be oversampled, though the extent to which these are affected is unclear. Nonetheless, the development of the RDS estimators sought to address potential over-representation of well-networked individuals by providing weights, which in essence down- or up-weight individuals’ data, based on their self-reported network size.[1, 5, 13] Such down-weighting was also apparent in our analysis of HIV infection and sexual identity where average network size was higher among subgroups of HIV infected MSM and those reporting homosexual identity, relative to other subgroups, and population prevalence estimates were consistently lower than sample proportions. While these RDS estimators have purported to produce unbiased estimates in other research, recent simulation studies and empiric research have demonstrated bias resulting from errors in self-reported network size and other potential sensitivities.[16, 18] These findings caution against over-reliance on use of RDS estimators and give credence to the inclusion of unweighted sample proportions along with population estimates produced by RDS-estimators.

Findings should be viewed in light of several limitations. First, we used unmodified research data from one setting and the generalizability of the demonstrated variability is unknown. We did not run simulations of the estimators, as such studies have already been conducted for other non-hidden populations and a few hidden populations to assess a range of issues and sources of bias.[6, 17, 18, 26, 28] This study, on the other hand, attempts to present how the estimators perform with natural variations in empirical data. None of the variables under study reached homophily greater than 0.42 and we were unable to demonstrate the potential influences of extremely high homophily. We may not fully identify limitations of the RDS estimators, as weaknesses have been noted when the sampling fraction is large, which it was not in this study.[17] While other studies and this one appear to highlight benefits of reaching greater recruitment depths, we did note that the 95%CI of the RDS estimators failed to include the sample proportion for several variables at high recruitment depths and the implications of this remain unknown. Finally, as with all RDS research to-date, there is no way to determine which estimator is correct.

Statistical software also plays a role in the decision of the estimator. RDSAT is a standalone, open access software that has been widely taught and is easily implemented. RDSAT, however, does not allow for additional analysis, whereas the RDS weights can be easily calculated and implemented within regression analyses when such analyses are performed using Stata or RDSA (in R, which is also open access). There is still no consensus, however, for how and whether RDS estimates should be incorporated into regression analyses to estimate magnitudes of associations within RDS samples.[2, 12] With respect to population prevalence estimates, our study demonstrated that there are no substantial differences across estimators after an appropriate recruitment depth has been reached. As previously reported, formative research is critical to informing study-specific decisions related to RDS and well as to identifying seeds who represent a range of characteristics to reduce potential seed bias in subsequent inference.[2, 17] Given current debates, it is recommended that several key pieces of information are included in all peer reviewed publications and surveillance reports: reporting of the selected RDS estimator along with the statistical program used to calculate weights and proportions, samples size, sample proportion and recruitment depths.[15, 16, 29] These recommendations are also highlighted, amongst others, in the recently released Strobe-RDS.[29] In the evolving development of RDS estimation methods, providing this information supports future use and critical review of research findings that are drawn from RDS methodologies.
